# CSE1L/CAS regulates cell proliferation through CDK signalling in mouse spermatogenesis

**DOI:** 10.1111/cpr.13334

**Published:** 2022-09-13

**Authors:** Jianwu Shi, Feng Qiao, Mei Ye, Ting Jiang, Jianni Liu, Mengya Zhang, Gangcai Xie, Kin Lam Fok, Xiaofeng Li, Hao Chen

**Affiliations:** ^1^ Institute of Reproductive Medicine Medical School of Nantong University Nantong China; ^2^ Medical Laboratory Department of Xiangyang Central Hospital Affiliated Hospital of Hubei University of Arts and Science Xiangyang China; ^3^ School of Biomedical Sciences, Faculty of Medicine The Chinese University of Hong Kong Hong Kong China; ^4^ Department of Laboratory Medicine Peking University Shenzhen Hospital Shenzhen China

To the editor

The cellular apoptosis susceptibility gene (CAS) (also named chromosomal segregation 1 like [CSE1L] and exportin‐2) has been found to play crucial roles in cell proliferation/apoptosis and progression of various cancers. While the functions of CAS in reproduction have not been well understood, previous studies in human trophoblast cells and seminoma showed that CAS is involved in cell proliferation and mitosis.^1^, ^2^ Our previous study demonstrated the expression of CAS in human testis and testicular cancers.^3^ We, therefore, speculate that CAS may exert its effect on spermatogenesis.

The process of spermatogenesis is complicated and tightly regulated. The sequential differentiating germ cells were produced during the mouse testicular development. Undifferentiated spermatogonia begin to differentiate 4 days postpartum (dpp). The spermatocytes come up in 10dpp, and the meiosis was accomplished at 20–21dpp. The spermatozoa was found in the 35dpp testis. The mice are sexual maturation at 56dpp. Therefore, we collected several key time points of the spermatogonia, spermatocytes, round spermatids, and spermatozoa to illustrate the expression profile of CAS during mouse testicular development. qPCR results showed that CAS mRNA was expressed at all stages of testicular development from 7 to 56 days postpartum (dpp) in the development‐dependent manner (Figure 1A). Similarly, the protein expression of CAS was also increased during the mouse testicular development (Figure 1B). To further determine the locations of CAS in the testes, the immunofluorescent staining was carried on at the crucial mouse testicular development point. As shown in Figure 1C, the signals of CAS were detected in spermatogonia in day 7 testes. The stronger signal of CAS was observed in day 21 testes in the spermatocytes and enriched in the nuclear and cytoplasm after day 35 testes. Of note, the sperm were no obvious staining of CAS (Figure S1). Consistent with the results of Figure 1C, the CAS was highly expressed in spermatogonia, spermatocytes and round spermatid (Figure 1D,E) in the published scRNA‐seq dataset.^4^

**FIGURE 1:**
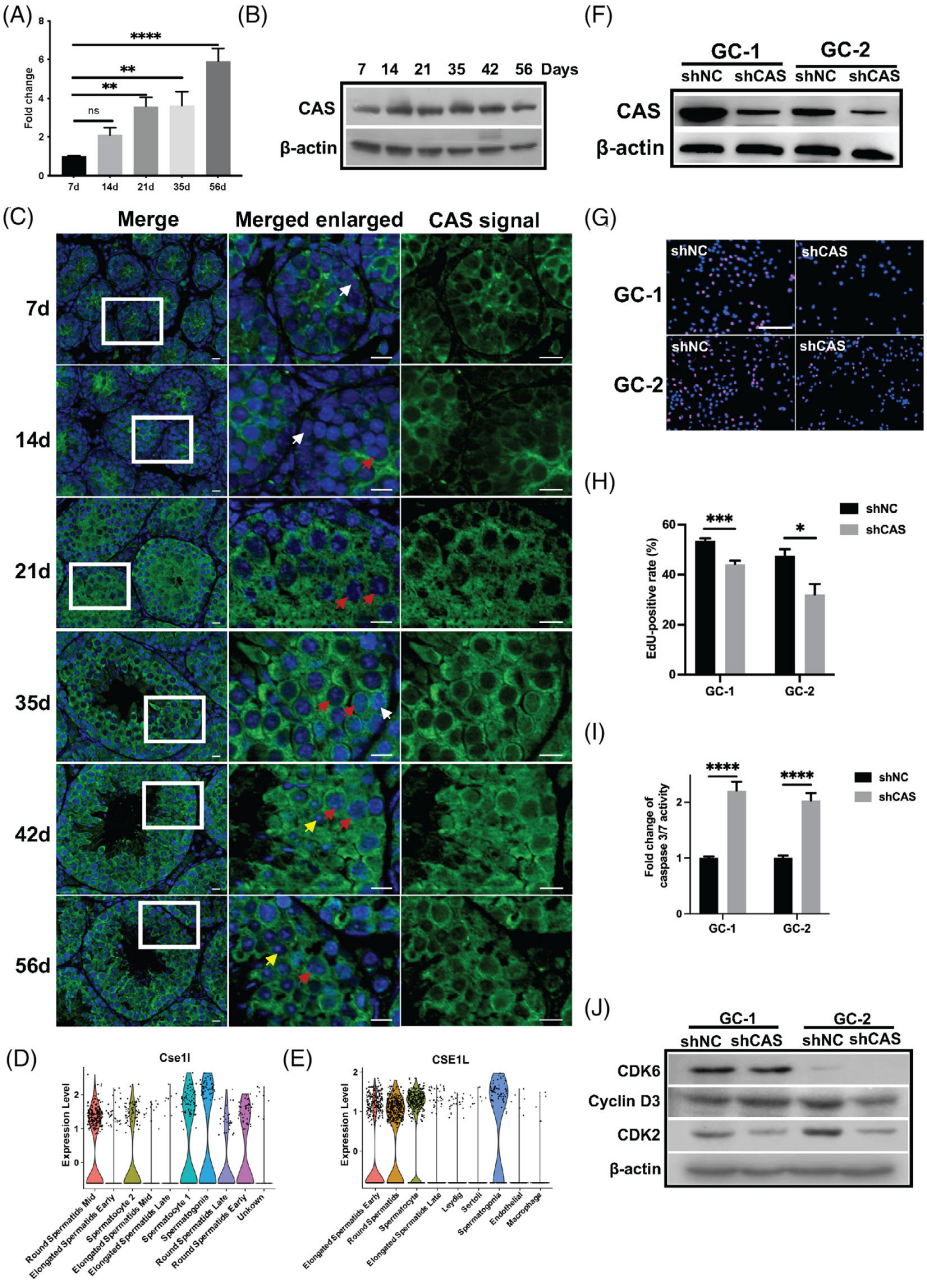
Expression profile of CAS during mouse testicular development and knockdown of CAS inhibited cell proliferation via decreased cell cycle‐related genes. (A) The mRNA expression level of CAS in developing mouse testis. **p < 0.01; ****p < .0001. (B) Representative Western blot of CAS proteins in developing mouse testis. (C) Representative pictures of CAS location in mouse testis development. CAS reactive signal was found in spermatogonia (white arrowhead), spermatocytes (red arrowhead) and round spermatid (yellow arrowhead). Scale bar = 10 μm. (D and E) Public single‐cell sequencing (scRNA‐Seq) results showed the expression of CAS in the different cell types in mouse (D) and human (E) testis. scRNA‐seq dataset GSE ID: GSE125372. (F) Western blot analysis of CAS knockdown in GC‐1 and GC‐2 cells. (G) Representative images of the EdU incorporation assay 96 h post‐transfection. Scale bar = 20 μm. (H) Statistical analysis of GC‐1 and GC‐2 cell proliferation as evaluated by an EdU incorporation assay. The values are presented as the mean ± SEM (*p < 0.05; ***p < 0.001). (I) Cell apoptosis analysis by caspase 3/7 activity assay kit (****p < 0.0001). (J) Western blot analysis of expressions of cell cycle‐related genes in CAS knockdown cells. CDK6, cyclin‐dependent kinase 6; CDK2, cyclin‐dependent kinase 6

Several studies have been demonstrated that CAS regulated the cell migration, proliferation and apoptosis in cancer cells.^5^, ^6^ However, the roles of CAS in germ cells proliferation are still unclear. To clarify this question, we employed a well‐established shRNA knockdown system used in our previous study^7^, ^8^ to knockdown the expression of CAS in spermatogonia cell line GC‐1 and spermatocyte cell line GC‐2 (Figure 1F and Figure S2A,B). The EdU incorporation assay revealed that knockdown of CAS resulted in a significant reduction of cell proliferation in GC‐1 and GC‐2 cells (Figure 1G,H). In addition, to elucidate the possible involvement of apoptosis elicited by interfering CAS, we determined the activity of caspase 3 and caspase 7 by the Caspase‐Glo 3/7 kit. As shown in Figure 1I, the activity of caspase 3/7 showed a remarkable increase in GC1‐shCAS and GC2‐shCAS cells compared with that of the GC1‐shNC and GC2‐shNC control group, suggesting that the knockdown of CAS was able to induce the cell apoptosis both in GC‐1 and GC‐2 cells. Our previous study has found the cell cycle arrest in the CAS knockdown breast cancer cells,^8^ with the observation that knockdown of CAS inhibited proliferation and induced apoptosis in GC‐1 and GC‐2 cells, we further explored the possible mechanisms underlying this phenomenon. Two markers of cell cycle CDK6 and CDK2, were significantly decreased after CAS knockdown in GC‐1 cells, while the CDK6, cyclin D3 and CDK2 were reduced after CAS knockdown in GC‐2 cells by western blot (Figure 1J and Figure S2C,D). Similar to the previous study, the migration ability of GC‐1 and GC‐2 was also blunted in the CAS knockdown group compared to the control group (Figure S3).

In summary, the present study for the first time demonstrated the expression patterns of CAS during mouse testicular development. Knockdown of CAS resulted in the inhibition of proliferation and migration of immortalized spermatogonia and spermatocytes, GC‐1 and GC‐2 cells, suggesting its potential role during spermatogenesis. Cyclin‐dependent kinases (CDKs) are the central regulators in the cell cycle. Several studies including ourselves demonstrated that CAS was involved in the proliferation/apoptosis via regulating factors of cell cycle.^1^, ^8^, ^9^ Interestingly, CDK6 and CDK2, but not cyclin D1 and B1 in breast cancer cell,^8^ were significantly inhibited in the CAS knockdown GC‐2 cells. Of note, CDK2 was reported to be essential for the first meiotic division in germ cells,^10^ together with our results, indicating that the role of CAS in regulating cell proliferation and apoptosis is tissue/cell‐specific.

It should be noted, however, that the mouse sperms showed weak immuno‐signal of CAS (Figure S1), indicating the turnover of CAS during spermiogenesis. In addition, the conditional knockout mouse models were warranted to be established due to the embryo lethal of knockout of CAS.^11^ Further investigation of these is undertaken in the authors' laboratory. Taken together, our results provide a novel role of CAS in spermatogenesis and a potential pathogenesis and diagnosis marker for male infertility.

## AUTHOR CONTRIBUTIONS

Hao Chen conceived and supervised the project. Jianwu shi, Feng Qiao, Mei Ye, Jianni Liu, Ting Jiang, Mengya Zhang and Gangcai Xie performed the experiments and analysed the data. Jianwu Shi, Feng Qiao, Xiaofeng Li and Kin Lam Fok wrote the manuscript. Hao Chen, Kin Lam Fok and Xiaofeng Li revised the manuscript.

## CONFLICT OF INTEREST

The authors declare that there are no conflicts of interest.

## Supporting information


**Figure S1** Immunostain of mouse sperm. The weak expression of CAS in mouse sperm. Red: CAS; Green: PNA; Blue: DAPI. Red arrowed: the head of sperm. Scale bar = 20 μm.

**Figure S2** (a and b) mRNA level of CAS knockdown in GC‐1 and GC‐2 cells.*p < 0.05. (c and d) The statistical analysis of protein expression level of CDK6, CDK2, and CyclinD3 in GC‐1 GC‐2 cells after CAS knockdown.

**Figure S3** CAS knockdown led to the inhibition of cell migration. Migration assay for GC‐1 and GC‐2 cells after CAS knockdown examined by both transwell assay (a–d) and wound healing assay (e‐h). *p < 0.05; ****p < 0.0001.

**Appendix S1** Supporting Information

## Data Availability

Data sharing is not applicable to this article as no new data were created or analyzed in this study.
