# Supplementary figures and images for: CSE1L/CAS regulates cell proliferation through CDK signalling in mouse spermatogenesis
Source: Cell Prolif. 2022 Sep 13;55(11):e13334. doi: 10.1111/cpr.13334 (PMC9628239; doi:10.1111/cpr.13334)

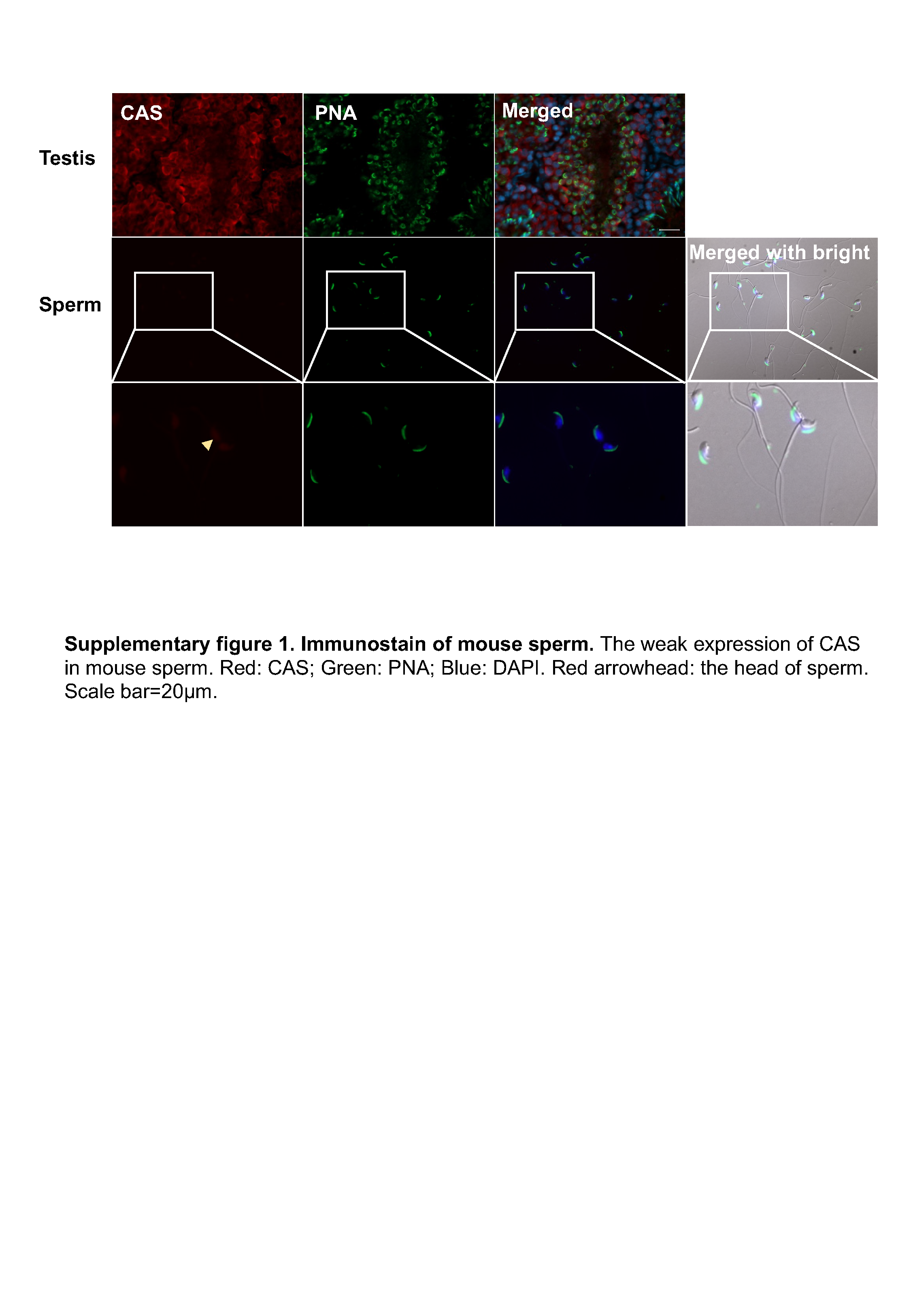

Supplement: Supplementary file 1 — Figure S1 Immunostain of mouse sperm. The weak expression of CAS in mouse sperm. Red: CAS; Green: PNA; Blue: DAPI. Red arrowed: the head of sperm. Scale bar = 20 μm. [file CPR-55-e13334-s004.tiff]

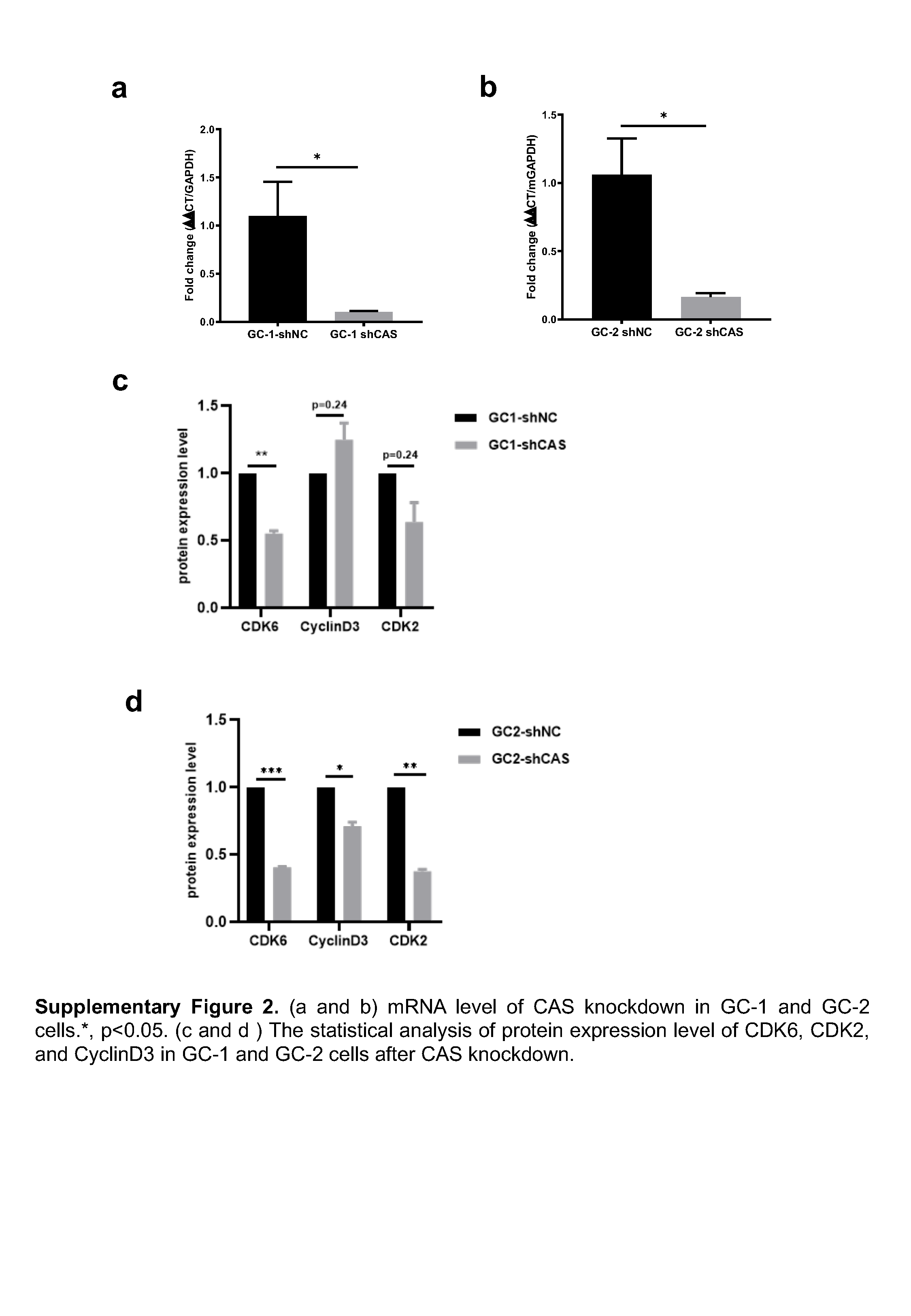

Supplement: Supplementary file 2 — Figure S2 (a and b) mRNA level of CAS knockdown in GC‐1 and GC‐2 cells.*p < 0.05. (c and d) The statistical analysis of protein expression level of CDK6, CDK2, and CyclinD3 in GC‐1 GC‐2 cells after CAS knockdown. [file CPR-55-e13334-s002.tiff]

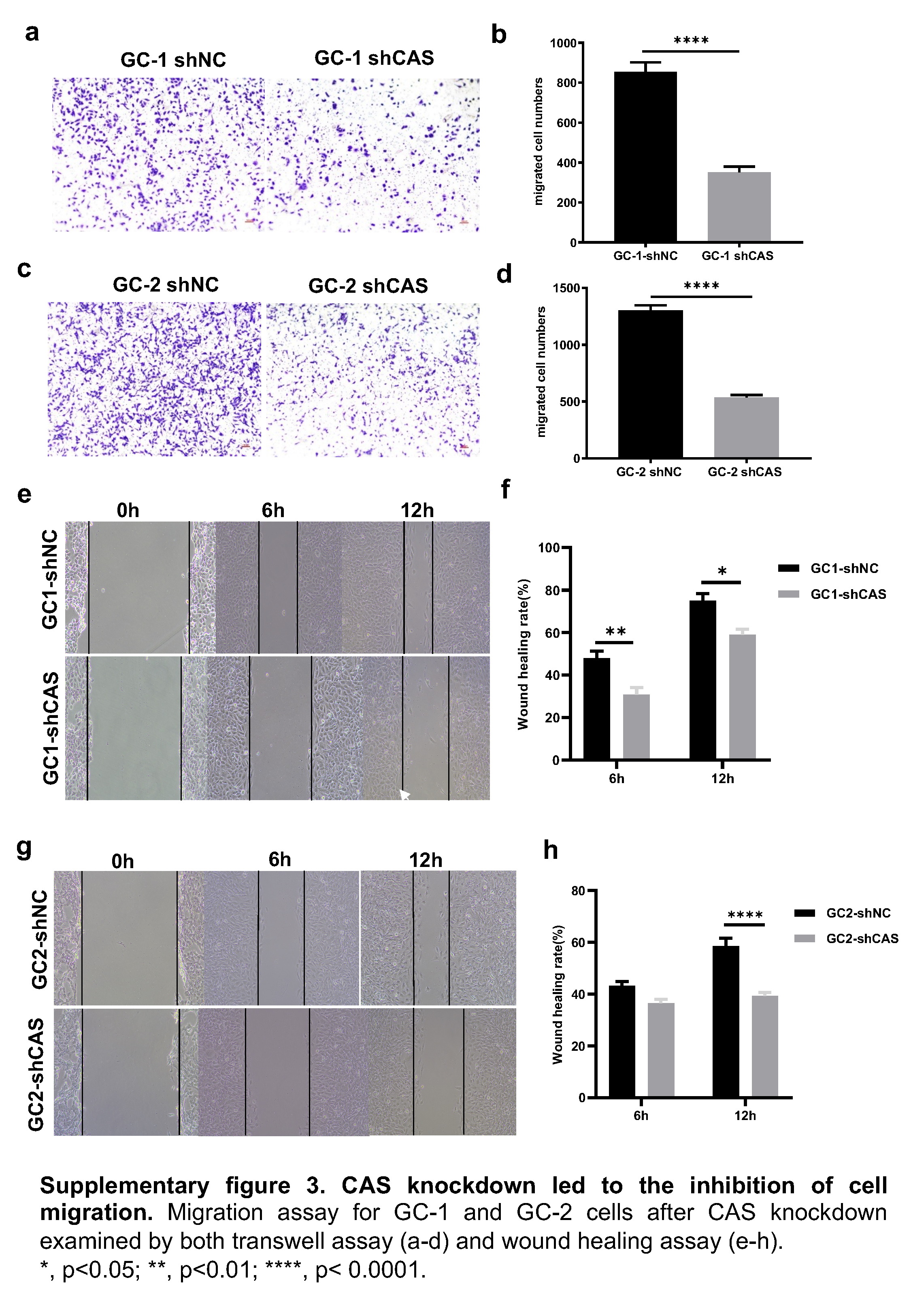

Supplement: Supplementary file 3 — Figure S3 CAS knockdown led to the inhibition of cell migration. Migration assay for GC‐1 and GC‐2 cells after CAS knockdown examined by both transwell assay (a–d) and wound healing assay (e‐h). *p < 0.05; ****p < 0.0001. [file CPR-55-e13334-s001.tiff]
