# Supplementary material for: CSE1L/CAS regulates cell proliferation through CDK signalling in mouse spermatogenesis
Source: Cell Prolif. 2022 Sep 13;55(11):e13334. doi: 10.1111/cpr.13334 (PMC9628239; doi:10.1111/cpr.13334)
Supplement: Supplementary file 4 — Appendix S1 Supporting Information [file CPR-55-e13334-s003.docx]

**Supplementary Materials and Methods**

**Animals**

Male C57BL/6J mice were bred in the SPF environment. All experiments were carried out under the license from the government of the Institutional Review Board of Nantong University.

**RNA isolation and qPCR analysis**

Testes were collected from different ages of male mice. Total RNA was extracted using Trizol reagent (TRI reagent, T9424, Sigma) according to the manufacturer’s instructions. qPCR assay was performed as described in our previous studies^1^. qPCR primers are as follows: Cse1l: F-GGATCAGGATCCAGGGTTTT; R-GAGTGGGGTAGTGTCGGCT; GAPDH: F-GACCACAGTCCATGCCATCACTGC; R-GCTGTTGAAGTCGCAGGAGACAAC.

**Immunofluorescence**

Testes were dissected from different ages of male mice and fixed in 4% paraformaldehyde. Testicular tissues were then dehydrated and embedded in the paraffin. The immunofluorescence assay was performed according to previous studies with minor modifications^2^. Briefly, 5μm-thick sections were used for the assay. After antigen retrieval, sections were incubated with 0.1 % TritonX-100 for 8 min and were blocked with blocking solution (5% donkey serum in 2% BSA) for one hour at room temperature. Then diluted primary antibody (CAS, ab27518, 1:500, Abcam) was added and incubated overnight at 4°C. After washing three times in PBST, the secondary antibody (Donkey anti-Rabbit IgG (H+L) Highly Cross-Adsorbed Secondary Antibody, Alexa Fluor 488, 1:500, Invitrogen) was incubated for 1 hour at room temperature. The nuclei were stained with 2 μg/mL DAPI for 5 min. All slides were mounted with an antifade mounting medium (ProLong™ Gold Antifade Mounting, P36970, Thermo Fisher) and then analyzed by fluorescence microscope.

**Cell culture**

GC-1 cell line (ATCC catalog No. CRL-2053) and GC-2 cell line (ATCC catalog No.CRL-2196) were preserved in our laboratory which were purchased from the American Type Culture Collection (ATCC). Those cell lines were cultured in Dulbecco’s modified Eagle’s medium supplemented with 10% fetal bovine serum and in 5% CO_2_ incubators at 37℃.

**Lentivirus transfection and western blot**

The lentiviral shRNA against Cse1l was purchased from Genepharma (Genepharma, Shanghai, China). The transfection procedures were performed as our previous studies described ^3^. Briefly, 1 × 10^5^ cells/well were plated into 6-well plates. After 12 h culture, the medium was replaced with fresh medium with lentivirus plus 0.1% polybrene. The expression of GFP was observed to determine transfection efficiency by fluorescence microscopy. Cells were lysed in RIPA lysis buffer after 48 hours of transfection. For the western blot analysis, 30 μg protein of total protein was loaded in SDS-PAGE and running with the Tris-glycine running buffer. The proteins were transferred onto PVDF membrane with the Tris-glycine transfer buffer in the condition of constant voltage 20V for 1h. The PVDF membrane was then incubated in the primary antibody overnight at 4°C and washed three times with TBST for 5min. After washing, the PVDF membrane was incubated with HRP-linked secondary antibody for 1h at room temperature and washed three times with TBST for 5min. Finally, the protein was detected by the enhanced chemiluminescence (ECL) method. The primary antibodies information are as follows: CAS (ab70547, 1:1000, Abcam), CyclinD3 (2936, 1:1000, CST), CDK6 (3136, 1:1000, CST), CDK2(2546, 1:1000, CST), β-actin (A2228, 1:5000, Sigma). The statistical analyses were calculated with Fiji^4^.

**EdU incorporation assay**

2 × 10^3^ cells were seeded in 96-well plates after 48h shRNA transfection. EdU was added into cells and incubated for 2 h at 37℃ CO_2_  incubator every 24h. After the incubation, we proceeded to the subsequent staining steps according to the kit instructions (Cell-Light EdU Apollo 567 In Vitro Imaging Kit, Ribobio). Fluorescence images were acquired by BioTek Cytation 1 system (Agilent Technologies).

**Migration assay**

Migration assay was performed as our previous study^3^. Briefly, after 48h shRNA transfection, 8 × 10^3^ cells were resuspended in the upper chambers with 400 μl serum-free DMEM and the lower chamber was added 600ul DMEM containing 10% FBS. After 24 h incubation, the cells that migrated into the lower chambers were counted and analyzed.

**Wound healing assay**

The wound healing assay was modified from the previously described method in our laboratory^5^. Briefly, 1.2 ×10^5^ cells were seeded in a 6-well plate. After the cell grew to a confluent monolayer, a scratch was made with a 10μl tip and the culture medium was changed into 2% FBS culture medium. Cellular migration was recorded using BioTek Cytation 1 system (Agilent Technologies)

Seven imaging views were calculated for each group.

**Caspase Activity Assay for Apoptosis Detection**

After 48h lentivirus transfection, cells were seeded in 96-well plates at a concentration of 2 × 10^3^ cells/well. Caspase-Glo 3/7 Reagent (G8090, Promega) was added to each well every 24 hours. After being gently mixed with a plate shaker, cells were incubated for one hour at room temperature. Finally, luminescence was measured in a BioTek microplate reader.

**Statistics**

GraphPad Prism 8 software was used to analyze the experimental data. Unpaired t test was used to analyze two group data. Ordinary one-way ANOVA was used to analyze significance in more than two groups. Multiple t test was used to evaluate two measurements. All data were presented as mean ± SEM.

1. Ye M, Han R, Shi J, et al. Cellular apoptosis susceptibility protein (CAS) suppresses the proliferation of breast cancer cells by upregulated cyp24a1. *Med Oncol.* 2020;37(5):43.

2. Shi J, Fok KL, Dai P, et al. Spatio-temporal landscape of mouse epididymal cells and specific mitochondria-rich segments defined by large-scale single-cell RNA-seq. *Cell Discov.* 2021;7(1):34.

3. Ye M, Chen Y, Liu J, et al. Interfering with CSE1L/CAS inhibits tumour growth via C3 in triple-negative breast cancer. *Cell Prolif.* 2022:e13226.

4. Schindelin J, Arganda-Carreras I, Frise E, et al. Fiji: an open-source platform for biological-image analysis. *Nat Methods.* 2012;9(7):676-682.

5. Chen H, Fok KL, Yu S, et al. CD147 is required for matrix metalloproteinases-2 production and germ cell migration during spermatogenesis. *Mol Hum Reprod.* 2011;17(7):405-414.
